# Smells like lemons: MYB-ADH gene cluster regulates citral biosynthesis in *Litsea cubeba*

**DOI:** 10.1093/plphys/kiad617

**Published:** 2023-11-17

**Authors:** Yana Kazachkova

**Affiliations:** Assistant Features Editor, Plant Physiology, American Society of Plant Biologists; Department of Molecular Biology, Princeton University, Princeton, NJ 08544, USA

Plants, among the planet’s best biochemists, possess the remarkable ability to synthesize millions of small molecules. Their astonishing chemical diversity plays an essential role in plant adaptation, defense, and interaction with the world around them. Terpenoids, the largest class of plant small molecules, play important roles in physiological and biochemical processes such as photosynthesis and phytohormone production. Some terpenoids have more specialized roles as pollinator attractants or defense compounds, and their biosynthesis is restricted to specific plant families (Pichersky and Raguso 2018; Zhou and Pichersky 2020).

The Lauraceae plant family includes trees, shrubs, and vines that are used for fruit, spice, and essential oils production. Citral is a monoterpene with a lemony aroma that is commonly found in Lauraceae species. Citral is used to enhance the flavor and fragrance of a wide range of products, including perfumes, soaps, food, and beverages. Citral consists of 2 monterpenes, α-citral (geranial) and its trans-isomer β-citral (neral), which are synthesized from geraniol and nerol, respectively. Alcohol dehydrogenase enzymes (ADHs) were previously shown to catalyze the conversion of geraniol to citral in sweet basil (*Ocimum basilicum*), *Persicaria minor*, and *Perilla spp.*; however, the role of ADHs in citral biosynthesis was investigated using only recombinant proteins (Iijima et al. 2006; Sato-Masumoto and Ito 2014; Tan et al. 2018). Additionally, little was known about the regulation of citral biosynthesis in planta. In this issue of *Plant Physiology*, Yunxiao Zhao and colleagues describe the Lauraceae-specific citral biosynthesis gene cluster that contains 2 alcohol dehydrogenase genes, *ADH28* and *ADH29*, encoding the modifying enzymes, as well as the negative regulator of citral biosynthesis, transcription factor MYB44 (Zhao et al. 2023).

*Litsea cubeba*, a plant species in the Lauraceae family, is characterized by high citral content in the fruit peel. Because ADH enzymes were previously shown to play a role in citral biosynthesis, the authors started by identifying all *ADH* genes in the *L. cubeba* genome. These genes were categorized into 3 clades through phylogenetic analysis and gene structure examination. The *LcADH27-36* genes in the *CAD* (*Cinnamyl Alcohol Dehydrogenase*) clade were proposed to play a role in citral biosynthesis. The authors examined the chromosomal distribution of *ADH* genes and found 2 gene clusters, *LcADH28-29* and *LcADH31-34*, on chromosomes 4 and 1, respectively. The *LcADH28-29* cluster consists of 4 genes, namely a transcription factor, *MYB44*, 2 alcohol dehydrogenases, *ADH29* and *ADH28*, and a MORF-Related Gene, *MRG1*, encoding a reader of histone mofications (Fig).

**Figure.**
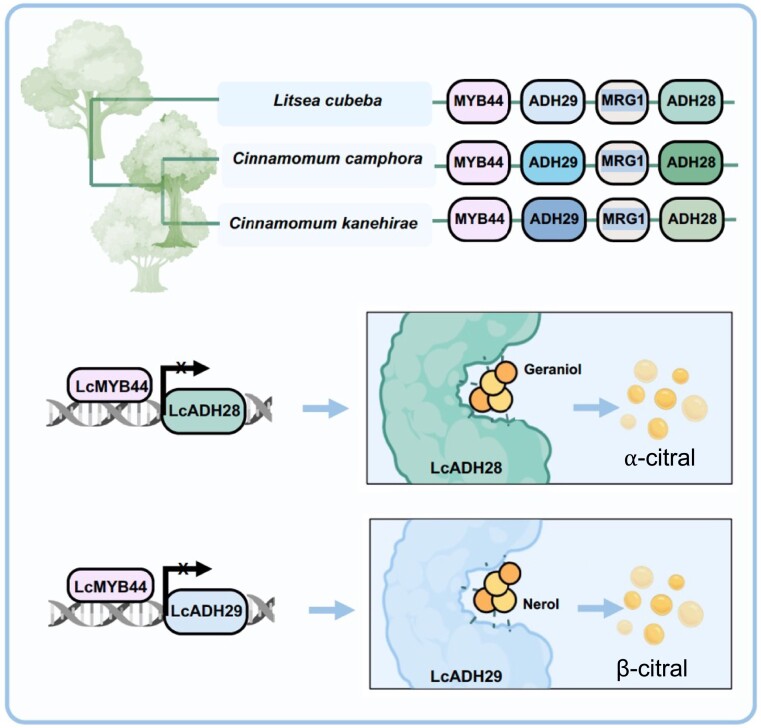
Schematic representation of Lauraceae family-specific citral biosynthesis gene cluster. Similar gene clusters were identified in 3 species in the Lauraceae family. The gene cluster contains MYB44 as a transcriptional regulator and 2 ADHs as modifying enzymes. Myb transcription factor LcMYB44 suppresses the gene expression of LcADH28 and LcADH29 genes by binding to their promoters (From Zhao et al. 2023). The 2 ADH enzymes show some divergence in substrate preference, with LcADH28 showing higher activity against geraniol and LcADH29 against nerol.

Zhao and colleagues investigated the evolution and divergence of *ADH* gene clusters in 3 related Lauraceae species: *Cinnamomum camphora*, *C. kanehirae*, and *Litsea cubeba*. Genomic analysis of these species, along with *Zea mays* and *Vitis vinifera*, revealed orthologs of *MYB44*, *MRG1*, *LcADH28*, and *LcADH29* in the Lauraceae species. To study citral biosynthesis in these species, *ADH28* and *ADH29* orthologs were cloned and expressed in *Escherichia coli* to test for in vitro enzymatic activity. Despite their high sequence similarity, *ADH28* and *ADH29* orthologs showed a divergence in their enzymatic activities and substrate preferences. LcADH29 exhibited a significant preference for nerol as its substrate, whereas LcADH28 had a higher affinity to geraniol.

To further study the role of *LcADH28* and *LcADH29* in citral biosynthesis, they were transiently expressed in *L. cubeba* leaves. The content of neral and geranial significantly increased with the increase in *LcADH28* and *LcADH29* gene expression. Transgenic *L. cubeba* lines overexpressing *LcADH2*8 and *LcADH29* were created and showed a significantly increased level of geranial. These results suggest that stable and transient overexpression of *LcADH28* and *LcADH29* can lead to increased accumulation of citral. Hence, the authors discovered 2 alcohol dehydrogenases, LcADH28 and LcADH29, that are important for citral accumulation in *L. cubeba*.

The authors next investigated the role of the other genes, *MYB44* and *MRG1*, in the citral biosynthesis gene cluster. Expression patterns of both *LcADH28* and *LcADH29* were consistent with citral accumulation levels in *L. cubeba* fruit; however, *MYB44* and *MRG1* showed a significant negative correlation with *LcADH* expression. This indicated a potential role of MYB44 as a negative regulator of citral biosynthesis. Indeed, in spearmint (*Mentha spicata*), MsMYB transcription factor was shown to downregulate monoterpene biosynthesis. Moreover, ectopic expression of *MsMYB* in basil and *Nicotiana sylvestris* affected the production of terpenoids (Reddy et al. 2017).

Dual-luciferase assays were conducted to investigate how LcMYB44 regulates the expression of *LcADH28*, *LcADH29*, and *LcMRG1*. The results showed that LcMYB44 acted as a repressor, reducing the expression of both *LcADH28* and *LcADH29*. Notably, LcMYB44 had a stronger inhibitory effect on *LcADH28* compared to *LcADH29*. Electrophoretic mobility shift assays using recombinant LcMYB44 protein and 48-bp promoter fragments of *LcADH28* and *LcADH29* revealed that LcMYB44 could directly bind to several sequences in their promoter regions, suppressing the gene expression. Suppression of *LcMYB44* expression in *L. cubeba* by virus-induced gene silencing led to increased expression of *LcADH28* and *LcADH29*, accompanied by enhanced accumulation of citral. Overexpression of *LcMYB44* resulted in decreased geranial and neral production. These results indicate that LcMYB44 is a negative regulator of citral biosynthesis that downregulates the expression of *LcADH28* and *LcADH29* genes. LcMYB44 did not activate the promoter of *LcMRG1*; therefore, further research is needed to understand the role of *LcMRG1* in the citral biosynthesis gene cluster.

In summary, in this study, Zhao and colleagues identified a Lauraceae-specific citral biosynthesis gene cluster containing 2 alcohol dehydrogenase enzymes, LcADH28 and LcADH29, and a transcription factor, LcMYB44, that negatively regulates *LcADHs* transcription and therefore decreases citral biosynthesis (Fig. 1). These results provide new insights into the evolution and regulatory mechanism of plant terpene biosynthesis.

## Dive Curated Terms

The following phenotypic, genotypic, and functional terms are of significance to the work described in this paper:

MYB44 Gramene: AT5G67300
MYB44 Araport: AT5G67300

alcohol CHEBI: CHEBI:30879

terpenoids CHEBI: CHEBI:26873

citral CHEBI: CHEBI:23316
